# Autologous Scleral Pocket Technique for Ahmed Glaucoma Valve Implantation with Pars Plana Tube Insertion for Neovascular Glaucoma

**DOI:** 10.3390/jcm10081606

**Published:** 2021-04-10

**Authors:** Hitomi Maki, Sotaro Mori, Hisanori Imai, Hiroko Yamada, Keiko Otsuka, Akiko Miki, Sentaro Kusuhara, Makoto Nakamura

**Affiliations:** Division of Ophthalmology, Department of Surgery, Kobe University Graduate School of Medicine, 7-5-2 Kusunoki-cho, Chuo-ku, Kobe 650-0017, Japan; makih047@med.kobe-u.ac.jp (H.M.); hisimai@med.kobe-u.ac.jp (H.I.); hiroky@med.kobe-u.ac.jp (H.Y.); keikoike1126@gmail.com (K.O.); acacyey@med.kobe-u.ac.jp (A.M.); kusu@med.kobe-u.ac.jp (S.K.); manakamu@med.kobe-u.ac.jp (M.N.)

**Keywords:** Ahmed glaucoma valve implantation, autologous scleral pocket technique, neovascular glaucoma, pars plana tube insertion

## Abstract

Specific postoperative complications, such as tube exposure and conjunctival erosion, have occurred despite the favorable surgical outcomes of tube shunt surgeries for refractory glaucoma. The new autologous scleral pocket technique is performed by inserting the tube into the vitreous cavity without using a donor scleral patch. The purpose of this study was to evaluate the surgical results of Ahmed glaucoma valve (AGV) implantation using this technique for neovascular glaucoma (NVG), which is one of the representative refractory types of glaucoma. This observational retrospective case series included 15 consecutive eyes of 15 patients with NVG who had undergone AGV implantation at Kobe University between January 2018 and December 2019. The mean preoperative intraocular pressure (IOP) was 37.2 ± 13.8 mmHg and the glaucoma drug score was 4.2 ± 2.2. The mean IOP and glaucoma drug score at 1 year postoperatively decreased to 15.0 ± 4.6 mmHg and 1.3 ± 2.0, respectively (*p* < 0.001). No significant change in the corneal endothelial cell density following surgery was observed (*p* = 0.09); however, one patient required an additional trabeculectomy at 7 months postoperatively. No cases of tube exposure or conjunctival erosion were observed at 1 year postoperatively. These results indicated the effectiveness and safety of this technique in patients with NVG.

## 1. Introduction

Neovascular glaucoma (NVG) is a catastrophic form of secondary glaucoma with a poor visual prognosis. It is characterized by elevated intraocular pressure (IOP), often associated with the presence of iris and/or iridocorneal angle neovascularization. Extensive ischemic retinal disorders, including proliferative diabetic retinopathy (PDR), retinal vein occlusion (RVO), and ocular ischemic syndrome (OIS), are the leading causes of NVG [1]. The treatment of NVG remains challenging; however, visual function can be preserved in some cases. The control of both elevated IOP and primary retinal disorders is essential for the treatment of NVG. To control the IOP, medical treatment is the first step in preventing the loss of vision; however, this frequently results in an insufficient IOP reduction, thereby necessitating surgical interventions, including trabeculectomy (TLE) and long tube shunt surgery (LTSS) [1].

Although TLE remains the first treatment modality for the surgical intervention of NVG, many recent studies have reported the usefulness of LTSS using the Ahmed glaucoma valve (AGV) or Baerveldt glaucoma implant [1,2,3,4,5,6,7,8,9,10] since the Tube Versus Trabeculectomy (TVT) study emerged [11]. Despite the satisfactory surgical outcomes of LTSS, several postoperative complications should be overcome; this includes common postoperative complications, such as endophthalmitis and the loss of corneal endothelial cells, as well as LTSS-specific postoperative complications, such as tube exposure and conjunctival erosion.

Exposure of the tube or plate and the erosion of the conjunctiva following LTSS is reported in 2–7% of the adult cases [2,3,7,9,11]—serving as a nidus for infection and a risk factor for endophthalmitis. To prevent these complications, patch grafts, including human donor sclerae [1,7,12,13,14,15], fasciae latae [16], dura mater [17], pericardia [15,18], corneas [19], autologous semithick scleral flaps [5,7,8,9,20,21], and autologous scleral tunnels [20,21,22,23,24], are usually used to cover the external portion of the tube. However, owing to the fragile structure of the frequently inflamed and surrounding scar tissue near the tube, placing the patch on top of the tube and closing the conjunctiva above this patch requires expertise and skill, especially when using a donor patch graft. Moreover, even after using these patch grafts, the tube may be exposed, and the conjunctiva may be eroded after surgery. Additionally, depending on the degree of cooperation with the donor bank, it may be difficult to obtain a donor patch graft.

Ozdamar et al. [22] first demonstrated the safety of the autologous scleral tunnel technique for AGV implantation into the anterior chamber without any donor patch grafts; subsequently, other groups also reported the safety of the autologous scleral flap and tunnel technique for AGV implantation into the anterior chamber [20,21,22,23,24]. These results suggest that LTSS does not require donor patch grafts; however, there were no reports examining whether the autologous scleral patch technique is also useful in LTSS using AGV with pars plana tube implantation. In this study, we evaluated the effectiveness and safety of the autologous scleral pocket technique in LTSS using AGVs with pars plana tube implantation for patients with NVG.

## 2. Materials and Methods

### 2.1. Patients

We retrospectively reviewed the medical records of 15 consecutive eyes of 15 patients with NVG who had undergone LTSS using AGV implantation at the Kobe University Hospital between January 2018 and November 2019. We excluded the second eyes of the patients who underwent binocular surgery as well as those who lacked a one-year follow-up after their surgical treatment. This study adhered to the tenets of the Declaration of Helsinki and was approved by the Institutional Review Board of Kobe University (No. 200091).

### 2.2. Surgical Procedure

**Figure 1:**
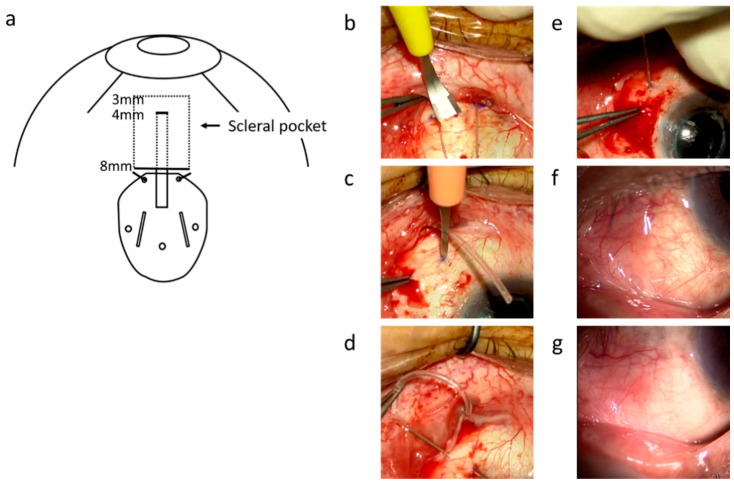
The surgical technique for tube insertion into the vitreous cavity through a scleral pocket. (**a**) is a schematic diagram of this surgery. The sclera was marked at 4 mm and 8 mm posterior to the limbus with a surgical pen. The episcleral suture for anchoring the AGV plate was placed 8 mm posterior to the limbus using 5-0 Dacron suture. The scleral pocket was made using a crescent knife (Mani, Tochigi, Japan) from 8 to 3 mm posterior to the limbus (**b**). The sclerotomy was created 4 mm posterior to the limbus with a side port straight knife (SP-S30, KAI, Tokyo, Japan) (**c**). The 27-gauge MaxGrip forceps (Alcon, Geneve, Switzerland) were passed through scleral pockets from the sclerotomy site, and the tip of the tube was grabbed with the forceps (**d**). The tube was inserted into the vitreous cavity through the sclerotomy site (**e**). Photographs of the anterior segment one month (**f**) and one year (**g**) after the surgery show smooth surface of the conjunctiva and absence of conjunctival dissociation and tube exposure.

This scleral pocket technique for AGV (FP-7^®^; New World Medical, Cucamonga, CA, USA) implantation was developed by one of the authors (H.I.; see Video S1, Supplemental Digital Content 1, which demonstrates the autologous scleral pocket technique for LTSS using AGVs) (Figure 1a). In brief, after topical and sub-Tenon anesthesia, a fornix-based flap of the conjunctiva (mainly lower nasal), created by limbal peritomy and Tenon’s capsule, was cut for AGV insertion. The sclera was marked at 4 mm and 8 mm posterior to the limbus with a surgical pen. The episcleral suture for anchoring the AGV plate was placed 8 mm posterior to the limbus using a double-armed 5-0 Dacron suture (Alcon, Fort Worth, TX, USA), and the scleral pocket was created using a crescent bevel-up ophthalmic knife (MCU26; Mani Inc., Utsunomiya, Tochigi, Japan) from 8 to 3 mm posterior to the limbus (Figure 1b). After the AGV was placed 8 mm posterior to the limbus, sclerotomy was performed 4 mm posterior to the limbus with a microsurgery knife (SP-S30; KAI, Seki, Gifu, Japan) (Figure 1c). Then, 27-gauge (27G) MaxGrip forceps (Griesharber^®^; Alcon) were passed through the scleral pockets from the sclerotomy site. The tip of the tube was grabbed with the forceps, inserted through the scleral pocket (Figure 1d), and pulled out from the sclerotomy site. The tube was adjusted to an appropriate length (4–5 mm) and then inserted into the vitreous cavity through the sclerotomy site (Figure 1e). After the tube’s location was confirmed using the scleral indentation technique, conjunctival suture was performed with an 8-0 vicryl suture (Ethicon, Somerville, NJ, USA) without using a donor scleral patch. Standard 27G pars plana vitrectomy (PPV) with a noncontact, wide-angle viewing system (Resight^®^; Carl Zeiss Meditec AG, Jena, Germany) was performed in the cases without a history of previous PPV, as well as to confirm peripheral vitrectomy or to perform additional panretinal photocoagulations (PRP) if necessary, prior to the LTSS procedure. All surgeries were performed at our clinic by three experienced vitreoretinal surgeons (A.M., H.I., and S.K.).

### 2.3. Ocular Biometrics

The IOP was measured five times in a row using noncontact tonometry (FT-1000; Tomey, Nagoya, Japan); the mean value was used as the IOP. A Landolt ring chart was used to measure the best-corrected visual acuity (BCVA); this was converted to the logarithm of minimal angle resolution (logMAR) for the statistical analyses. In this study, a visual acuity of 0.01 and the ability to count fingers were denoted as a logMAR of 2.0. Hand motion, light sense, and no light perception were scored as 2.9, 3.2, and 3.5, respectively [25]. Noncontact-type specular microscopy (Noncon Robo SP-8000; Konan Medical, Tokyo, Japan) was used to measure the endothelial cell density (ECD) in the central area of the cornea.

### 2.4. Data Analysis

The IOP and glaucoma drug scores were measured preoperatively and at 1 week and 1, 3, 6, and 12 months postoperatively. In terms of the glaucoma drug score, the combination of the eye drops and the oral carbonic anhydrase inhibitors scored 2 points. The BCVA was measured preoperatively and at 1 year postoperatively. The ECD was measured preoperatively and at 3 months postoperatively. The preoperative and postoperative values were compared using a mixed-effect model. SPSS® (version 24; IBM, Armonk, NY, USA) was used for the statistical analysis. The statistical significance was set at *p* < 0.05.

## 3. Results

Table 1 shows the preoperative demographics of the 15 patients. Among these patients, seven (47%) had a history of PPV, and two (13%) had a history of glaucoma surgery.

The underlying diseases of NVG were PDR in seven eyes (47%), RVO in six eyes (40%), and OIS in two eyes (13%). Thirteen (87%) eyes underwent vitrectomy concomitant with tube insertion (eight vitreous eyes (53%) and five avitreous eyes (33%)). Ten eyes (67%) underwent additional intraoperative PRP. PRP was performed in one eye with RVO without a preoperative retinal photocoagulation for the first time. Four eyes (27%) did not undergo additional photocoagulation. All four eyes that did not receive PRP had a history of PPV. Two phakic eyes underwent cataract surgery during the procedure.

**Figure 2:**
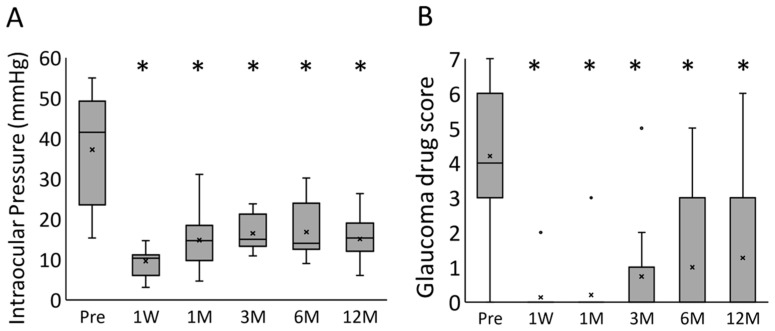
The box-and-whisker plots for the time course changes of the (**A**) intraocular pressure and (**B**) glaucoma drug score. * *p* < 0.0001 (mixed-effect model); Pre, preoperative; 1W, 1 week postoperatively; 1M, 1 month postoperatively; 3M, 3 months postoperatively; 6M, 6 months postoperatively; 12M, 12 months postoperatively.

Successive pre- to postoperative changes in the IOP and glaucoma drug scores in all 15 eyes are shown in Figure 2. The IOP and glaucoma drug score decreased significantly at all timepoints when compared with the preoperative values. The median IOP and glaucoma drug scores preoperation and at 1 week, 1 month, 3 months, 6 months, and 1 year postoperation were 41.5 mmHg and 4, 10.3 mmHg and 0, 14.7 mmHg and 0, 15.0 mmHg and 0, 14.0 mmHg and 0, and 15.3 mmHg and 0, respectively; both showed a significant decrease following the surgery (*p* < 0.0001, mixed-effect model).

**Figure 3:**
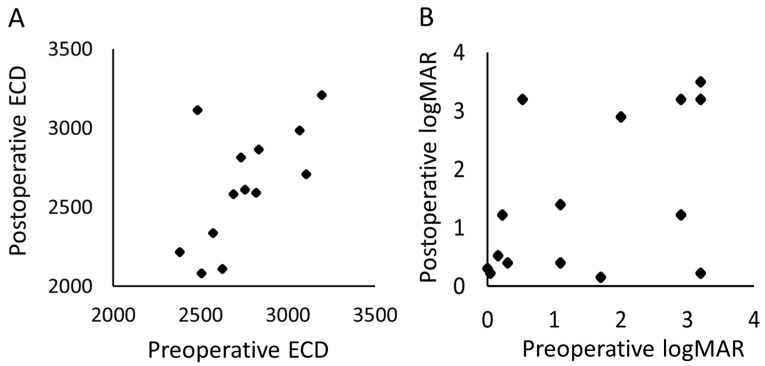
The scatter plots of the preoperative and postoperative (**A**) corneal endothelial cell density and the (**B**) logMAR. ECD, corneal endothelial cell density; logMAR, logarithm of minimal angle resolution.

Figure 3 shows a scatter plot of the VA and ECD between the preoperative and one-year postoperative periods. The ECD could not be measured in 1 eye postoperatively; hence, 14 eyes were compared. The mean (standard deviation) period of the postoperative ECD was 11.8 (5.4) months. There was no significant change in the postoperative VA when compared with the preoperative values (*p* = 0.93, paired *t*-test); similarly, the ECD did not show a significant decrease relative to the preoperative values (*p* = 0.087, paired *t*-test).

Table 2 shows the postoperative complications: although a vitreous hemorrhage was observed in one eye, no additional PPV was required for the cleansing. The cystoid macular edema in one patient was resolved by a topical, nonsteroidal, anti-inflammatory drug instillation for 3 months. One patient exhibited a persistently high postoperative IOP and underwent additional trabeculectomy 7 months postoperatively; however, despite these interventions, the patient unfortunately lost visual function, possibly due to glaucoma progression. There were no cases of tube exposure or conjunctival erosion up to 1 postoperative year.

## 4. Discussion

In the current case series, a significant reduction in the IOP was achieved following LTSS using AGVs combined with the autologous scleral pocket technique. The postoperative IOP following LTSS with AGVs is generally reported to be in the mid-teens range [11]. When focusing on the effect of LTSS on NVG, the average preoperative IOP was reported to be 30.9–49.9 mmHg (median, 42.8 mmHg) [1,2,3,4,5,6,7,8,9,10], which is remarkably high compared to the preoperative IOP for the other glaucoma types; however, the average postoperative IOP was reported to be 11.8–21.7 mmHg (median, 15.5 mmHg) [1,2,3,4,5,6,7,8,9,10], which is comparable to the posttreatment results for the other types of glaucoma. In this study, the pre- and postoperative IOPs were 37.2 mmHg and 15.0 mmHg, respectively. In terms of the glaucoma drug score, our results could decrease the use of hypotensive medications as previously reported [5,6,7,8,9]. Our results also preserved VA, assuming that the postoperative findings did not decrease significantly. These results suggest that LTSS using AGVs in combination with the autologous scleral pocket technique could achieve the same level of IOP reduction compared to previous reports.

No exposure of the tube or plate nor erosion of the conjunctiva was observed following the coverage of the AGV tube using the autologous scleral pocket in the current study. Ollila et al. [20] reported that no conjunctival complications were observed among 92 patients who underwent LTSS using a Molteno implant with the scleral tunnel technique, while conjunctival erosions occurred in 15 eyes (4.5%) covered only by the conjunctiva following an anterior chamber tube insertion. Another report also elucidated that LTSS using AGVs without any patch exhibited a high rate of tube erosion (12.9%; 11 of 96 eyes) following an anterior chamber tube insertion [13]. These results suggest that coverage of the tube using a patch is essential for preventing conjunctival erosion following LTSS; however, no conclusions regarding the optimal patch type have been reached.

Several previous reports demonstrated that the autologous scleral tunnel procedure resulted in better outcomes compared with donor sclera or pericardium patch grafts following an anterior chamber tube insertion. Tamcelik et al. showed that tube exposure was seen in 6 (2.2%) of 78 eyes with a donor scleral patch, but there was no exposure with 129 eyes using the autologous scleral tunnel technique after an anterior chamber tube insertion [13]. Another report presented 1 (2.5%) case of tube exposure among 40 cases using the autologous scleral tunnel technique, and 3 (7.9%) cases among 48 covered by a donor pericardium patch graft after an anterior chamber tube insertion [18]. Pakravan et al. conducted a randomized clinical trial that compared graft-free autologous short scleral tunnels and half-thickness scleral patch grafts in AGV implantation; the tube erosion was only observed in one (1.0%) case of the half-thickness scleral patch graft after the anterior chamber tube insertion [21]. These results suggest the superiority of autologous patch grafts, including scleral tunnels and flaps, compared with donor patch grafts. We also did not experience exposure of the tube or erosion of the conjunctiva following the pars plana tube insertions for one year using the autologous scleral pocket technique (autologous patch graft).

These findings suggest the effectiveness of our new technique. The reason for the better postoperative results with autologous patch grafts than with donor patch grafts is still unknown. We hypothesized that (1) the immune response toward the donor patch graft from the host could invoke a biological inflammatory response and result in the melting of the surrounding tissue, including the donor patch graft and the host conjunctiva; and (2) if the donor patch graft, which is an avascular tissue, does not engraft in the host, the tissue could collapse, resulting in conjunctival erosion and tube exposure. Focusing on the autologous scleral patch graft, Tamcelik et al. reported that conjunctival erosion following the autologous scleral flap technique could occur more frequently than after the autologous scleral tunnel technique [13]. This suggests that the frequency of conjunctival exposure may differ depending on the method of patch preparation, even in the case of an autologous scleral patch, including the scleral flap, tunnel, and pocket technique. We hypothesize that even with an autologous scleral patch graft, it is important to maintain the patch’s blood flow as close to normal as possible. When comparing the postoperative blood flow between the autologous half-thickness scleral flap technique (which requires cutting the three sides of the flap) and the autologous scleral tunnel technique (which requires cutting two sides of the sclera), the latter is expected to yield more blood flow to the scleral patch, as well as to maintain scleral health; this decreases the degree of the tissue’s collapse and the possibility of exposing the conjunctiva. Our autologous scleral pocket technique may have the advantage of preserving the patch’s blood circulation since we cut only on one side of the sclera, possibly contributing to the prevention of ocular inflammation and tube exposure. A further examination is essential in future studies.

It is also noteworthy that in this study, there was no decrease in the density of the corneal endothelial cells one year following surgery. Previous studies reported that when a tube is placed in the pars plana, the postoperative corneal endothelium loss is significantly suppressed compared with the anterior chamber placement [26,27]. By contrast, the possibility of complications such as postoperative vitreous hemorrhage and retinal detachment has also been reported following pars plana tube insertion [26,27]. In this report, there were cases in which postoperative complications such as vitreous hemorrhage occurred; thus, a complete peripheral vitrectomy was needed to prevent the tube’s obstruction by a vitreous body, even though this was not observed in this study. However, the density of the corneal endothelial cells was maintained. From this point of view, it is necessary to deliberate on the optimal location of tube insertion; however, in the case of vitrectomized eyes and the need to combine vitrectomy with PRP, pars plana insertion of the tube in combination with the autologous scleral pocket method could be a treatment option. Ciliary sulcus placement may be another option to prevent corneal damage; however, our recent study [28] and another group [29] demonstrated that although this technique was useful in decreasing the corneal endothelial density loss rate, this result was significantly reduced over time. We therefore consider that the placement of the tube in the vitreous cavity when avoiding corneal damage is a top priority. In the future, it would be necessary to increase the number of cases to be considered.

This study had several limitations. First, it was a single-institution study with a small sample size, which may lead to a noteworthy bias in our results. Second, some cases underwent an AGV implantation with a concomitant PPV or lensectomy; this additional procedure may also have contributed to the IOP reductions. Third, we followed patients for 1 year after the operation; thus, more longitudinal observations are needed. However, Ollilla et al. described 9 in 16 cases of conjunctival erosions within 10 months postoperatively, and 6 cases with the same complications occurred following the additional glaucoma surgeries [20]. Tamcelik et al. also documented 16 of 18 tube exposure cases that occurred 1 year following surgery [13]. These results suggest that conjunctival erosion and tube exposure are usually seen in the early postoperative period, within one year. Therefore, further research supporting our hypothesis of the pathological or blood flow parameters comparing the patch graft technique to our scleral pocket technique is warranted.

In conclusion, our autologous scleral pocket technique for LTSS using AGVs could be a treatment option for NVG, and could reduce postoperative conjunctival erosions and tube exposure.

## Figures and Tables

**Table 1:** The baseline clinical characteristics of the participants.

Age, Years	67.7 (11.3)
Sex	Male: 8 (53%) Female: 7 (47%)
Primary diseases of NVG	PDR: 7 (47%) RVO: 6 (40%) OIS: 2 (13%)
Post-vitrectomy	7 (47%)
IOP, mmHg	37.2 (13.8)
Glaucoma drug score	4.2 (2.2)
ECD (cells/mm^2^)	2687.1 (304.6)

The continuous variables are expressed as the mean (standard deviation). NVG, neovascular glaucoma; IOP, intraocular pressure; ECD, corneal endothelial cell density; PDR, proliferative diabetic retinopathy; RVO, retinal vein occlusion; OIS, ocular ischemic syndrome.

**Table 2:** The postoperative complications.

Vitreous Hemorrhage	6 (40%)
Hyphema	4 (27%)
Choroidal detachment	3 (20%)
Macular edema	1(7%)
Iris posterior synechia	1 (7%)
Hypotony maculopathy	1 (7%)
Additional glaucoma surgery	1 (7%)
Tube erosion, conjunctival erosions	0 (0%)

## Data Availability

The data that support the findings of this study are available from the corresponding author, S.M., upon reasonable request.

## Supplementary Materials

The following are available online at https://www.mdpi.com/article/10.3390/jcm10081606/s1, Video S1: Video that demonstrates autologous scleral pocket technique for LTSS using AGV.
